# Bovine Tuberculosis at the Wildlife-Livestock-Human Interface in Hamer Woreda, South Omo, Southern Ethiopia

**DOI:** 10.1371/journal.pone.0012205

**Published:** 2010-08-17

**Authors:** Rea Tschopp, Abraham Aseffa, Esther Schelling, Stefan Berg, Elena Hailu, Endalamaw Gadisa, Meseret Habtamu, Kifle Argaw, Jakob Zinsstag

**Affiliations:** 1 Swiss Tropical and Public Health Institute, Basel, Switzerland; 2 Armauer Hansen Research Institute, Addis Ababa, Ethiopia; 3 Veterinary Laboratories Agency, Addlestone, United Kingdom; 4 Ethiopian Wildlife Conservation Authority, Addis Ababa, Ethiopia; McGill University, Canada

## Abstract

Bovine tuberculosis (BTB) is endemic in cattle in the Ethiopian Highlands but no studies have been done so far in pastoralists in South Omo. This study assessed the prevalence of bovine tuberculosis (BTB) at an intensive interface of livestock, wildlife and pastoralists in Hamer Woreda (South Omo), Ethiopia. A cross-sectional survey including a comparative intradermal skin testing (CIDT) was conducted in 499 zebu cattle and 186 goats in 12 settlements. Sputum samples from 26 symptomatic livestock owners were cultured for TB. Fifty-one wildlife samples from 13 different species were also collected in the same area and tested with serological (lateral flow assay) and bacteriological (culture of lymph nodes) techniques. Individual BTB prevalence in cattle was 0.8% (CI: 0.3%–2%) with the >4 mm cut-off and 3.4% (CI: 2.1%–5.4%) with the >2 mm cut-off. Herd prevalence was 33.3% and 83% when using the >4 and the >2 mm cut-off respectively. There was no correlation between age, sex, body condition and positive reactors upon univariate analysis. None of the goats were reactors for BTB. Acid fast bacilli (AFB) were detected in 50% of the wildlife cultures, 79.2% of which were identified as *Mycobacterium terrae* complex. No *M. bovis* was detected. Twenty-seven percent of tested wildlife were sero-positive. Four sputum cultures (15.4%) yielded AFB positive colonies among which one was *M. tuberculosis* and 3 non-tuberculous mycobacteria (NTM). The prevalence of *M. avium*-complex (MAC) was 4.2% in wildlife, 2.5% in cattle and 0.5% in goats. In conclusion, individual BTB prevalence was low, but herd prevalence high in cattle and BTB was not detected in goats, wildlife and humans despite an intensive contact interface. On the contrary, NTMs were highly prevalent and some *Mycobacterium* spp were more prevalent in specific species. The role of NTMs in livestock and co-infection with BTB need further research.

## Introduction

Bovine tuberculosis (BTB) is a chronic infectious disease caused by *Mycobacterium bovis*, a pathogen closely related to *M. tuberculosis*, and member of the *Mycobacterium tuberculosis* complex (MTC) [1].

Eradicated or controlled in most parts of the developed world, BTB remains prevalent in Sub-saharan African countries, where national control strategies are often non-existent [2]. Besides being a potential zoonotic threat through consumption of raw animal products and close animal-human contact, the disease can have major economic impacts on national livestock sectors [3]. BTB can also circulate in wildlife, fuelling new outbreaks in livestock at the livestock-wildlife interface, thus hampering costly national control programs [4], [5]. In addition, the disease can be a serious risk for endangered wildlife species [6].

BTB is endemic in the cattle population of the Ethiopian Highlands but the prevalence varies by region depending on prevailing breed (exotic taurin breeds versus local zebu breeds) and farming practice. High prevalence [7.9% to 78.7%] was found in peri-urban and/or urban areas, which are characterized by high numbers of dairy farms, exotic breeds and their crosses kept under intensive or semi-intensive husbandry systems [7]–[10]. In contrast, low BTB prevalences [0–2.4%] were found in cattle among agro-pastoralist small holders in rural areas in the Highlands, where they keep zebu cattle in smaller numbers under traditional management system [11]–[13].

While most studies focused on the Highlands, very little data on BTB is currently published from the lowland areas in Ethiopia that also include mobile pastoralists. It is often speculated that diseases such as BTB are prevalent in nomadic populations due to their lifestyle, herd size and environment/climate, but very few data on BTB exist from these communities in general and from nomadic pastoralists in Ethiopia in particular. The remoteness of sites, the difficult logistics involved and/or poor security in these areas are contributing factors to the scarcity of research studies.

To date, the few published reports from these communities include an abattoir study in Borana (Southern Ethiopia) by Demelash et al (2009) [14] who described a BTB prevalence of 4.2%. Similarly in the same region, Gumi (2009) [15] described a field prevalence of individual tuberculin reactors of 5%.

The South Omo zone located in the Southern Ethiopian lowlands has one of the highest cattle densities per 1000 inhabitants in the country [16]. Within the zone, Hamer Woreda is inhabitated mainly by the Hamer, a pastoralist ethnic group moving seasonally with their herds in search of grazing land and water, whereas the Karo tribe, few in numbers nowadays (less than 3000), have partly become agro-pastoralists using the fertile banks of the Omo river. Pastoralists keep large number of livestock, in particular cattle, which are the core of their socio-economical life, their culture and their daily survival. Most of their protein intake is covered by raw milk and blood consumption from cattle as well as goat meat.

The lower Omo Valley has a large and diverse range of wildlife species [17]. Tribal warfare is common in the area and has allowed wildlife to thrive along ethnic territorial boundaries [18]. However the growing human and livestock population in addition to recurrent droughts puts increasing pressure on these habitats, thus intensifying the human-livestock-wildlife interface in the region and more particularly in and around national parks and/or control hunting areas such as Murule where pastoralist livestock have been observed to share same grazing land as wildlife.

This study investigates BTB prevalence in livestock, wildlife and pastoralists at the contact interface in the West of Hamer Woreda in and around the Murule controlled hunting area. To our knowledge, this is the first study of bovine tuberculosis in the South Omo Zone.

## Materials and Methods

### Study site

**Figure 1:**
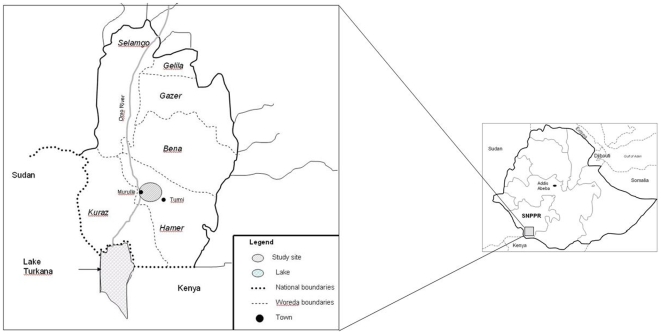
Map of study site where livestock, wildlife and human samples were collected for BTB investigation.

The study was carried out in the Hamer Woreda within the South Omo Zone, which is part of the Southern Nations, Nationalities and Peoples Region (SNNPR) (Figure 1). The centre of the study area was located 5.1°N and 36.1°E and had an average elevation of 420 meters above sea level. The study was carried out in the Murule Controlled Hunting Area and 12 pastoralist settlements within or adjacent to this area. The sites were all located on the Eastern bank of the Omo River, which flows south into Lake Turkana and were inhabited by semi-mobile pastoralist groups of which the most important ones were the Hamer. One settlement was inhabited by a pastoralist Karo tribe.

### Study design

A cross-sectional study of BTB in livestock was conducted, in which the settlements were considered as clusters. Livestock owners kept their cattle and their goats in separate enclosures at night for protection against predators and theft. The authors considered each individual animal from individual owners when calculating individual BTB prevalence. However, when calculating BTB herd prevalence, the authors did not consider livestock belonging to one owner herd but regrouped all cattle of all owners in one settlement herd, respectively all goats (that were analyzed separately) since livestock was kept communally during the day and thus constituting a natural epidemiological unit.

A list of settlements in the area was obtained from the Hamer Woreda agricultural office. Inclusion criteria for settlements were: logistical feasibility (accessibility by car; security; distance), proximity between settlements and wildlife areas, and cooperation of pastoralists (after first informing in details the elders of each settlement about the research, they gave their consent that the settlement participates in the study). Random selection procedure of settlements was not always feasible due to inclusion criteria. Cattle and goats were selected randomly within herds.

Wildlife were harvested in the Murule Controlled hunting area by professional hunters under license, in accordance to official quotas (species and numbers) set by the Ethiopian Wildlife Conservation Authority.

### Study animals

Cattle and goats were tested in the settlements of the study area between May 2007 and March 2009. Visits to settlements were made early morning or late afternoon when animals were kept in their enclosures. Testing of animals was performed after informed verbal consent was given by the individual owners. Calves or goats younger than 6 months of age, peri-parturiant animals, and animals showing any clinical symptoms of infectious diseases were not included in the study.

Information on sex, age, and body condition scores was collected for each tested animal, and for cattle also type of breed. Cattle were categorized into four age groups: calves between six month and one year old, juveniles between one and three years, breeders between three and ten years and animals older than ten years. Body condition scoring of the animals was done using a 1 to 5 scale, where score 1 was representing emaciated and score 5 fat animals [19]. For the final data analysis, scoring data was regrouped into 3 categories (1: emaciated/thin; 2: normal; 3: muscoulous/fat). Cattle were differentiated in three different types of breed: traditional zebus, cross-breeds and exotic breeds. Within the traditional zebu breeds, animals were if possible, further categorized based on phenotypical characteristics. Sheep were of local nondescript breed. Various wildlife species were sampled in the area (Table 1).

**Table 1:** Serological and culture results of investigated wildlife in Hamer Woreda, South Omo.

Species	Total number animals (serology; culture)	Serology positive (%)	Culture positive (%)	Culture result
Grant's gazelle (*Nanger granti*)	17 (12; 17)	4 (33.3)	8 (47)	MTC, MAC**, *M.wollinskyi, M.asiaticum*
Lesser Kudu (*Tragelaphus imberbis*)	7 (5; 7)	3 (60)	2 (28.6)	MTC, *M.flavescens*
Guenther's dik-dik (*Madoqua gunther*)	7 (3; 7)	1 (33.3)	3 (43)	MTC
Gerenuk (*Litocranius walleri*)	5 (4; 5)	0 (0)	4 (80)	MTC, Nocardia spp.
Tiang (*Damaliscus lunatus*)	4 (3; 4)	0 (0)	2 (50)	MTC
Hartebeest (*Alcelaphus buselaphus*)	4 (3; 2)	0 (0)	1 (50)	MTC
Greater Kudu (*Tragelaphus strepsiceros*)	1 (ND; 1)		1 (100)	MTC
Bushbuck (*Tragelaphus scriptus*)	1 (ND; 1)		1 (100)	MTC*
Waterbuck (*Kobus ellipsiprymnus*)	1 (1; 1)	1 (100)	0 (0)	
Buffalo (*Syncerus caffer*)	1 (1; ND)	0 (0)		
Anubis baboon (*Papio anubis*)	1 (ND; 1)		0 (0)	
Serval cat (*Leptailurus serval*)	1 (ND; 1)		1 (100)	MTC, *M.gilvum*
Black-backed jackal (*Canis mesomelas*)	1 (1; 1)	0 (0)	1 (100)	MTC
Total	51 (33; 48)	9 (27)	24 (50)	

*MTC: Mycobacterium terrae complex.

**MAC: Mycobacterium avium complex.

### Skin testing of livestock

We applied the comparative intradermal tuberculin test (CIDT) in all cattle and goats using both avian and bovine purified protein derivates (PPD) supplied by the Veterinary Laboratories Agency, Weybridge, UK. Intradermal injections of 0.1 ml (2,500 IU/ml) bovine PPD and 0.1 ml (2,500 IU/ml) avian PPD were made in two shaved sites. In cattle, the two shaved sites were 12 cm apart from each other in a horizontal line on the same side of the middle neck region. In goats, a site was shaved on both sides of the middle neck area. Skin thickness was measured with a caliper prior to injections and again at both injection sites after 72 hours. The reaction at each site was calculated as the difference of the skin thickness after 72 hours minus before injection. Data was analyzed using two definitions of positive reactor: 1) an animal was considered BTB positive if the reaction at the bovine site minus the reaction at the avium site was greater than 4 mm (official OIE definition), 2) an animal was considered positive if the bovine site minus the reaction at the avium site was greater than 2 mm [20], [21].

The authors defined arbitrarily *Mycobacterium Avium* Complex (MAC) positive animals as the difference of the skin fold on the avium site being greater than 4 mm at 72 hours after injection.

### Wildlife sampling and laboratory analysis

Wildlife tissue and blood were collected by professional hunters in the Murule controlled hunting area between 2006 and 2008. They collected mediastinal, mesenteric, and submandibular lymph nodes as well as “tuberculous-like” organ lesions from fresh carcasses and filled in a sample collection form to record date and place of sample collection, information on the species, and information on organs collected. All sample specimens were kept in phosphate buffer saline (pH 7.2). Tubes with sampled blood were stored upright for 6–12 hours, sera pipetted and transferred into 1 ml cryo-tubes. All tissue and sera samples were kept cool (4°C) on site, transported in cool-boxes to the Armauer Hansen Research Institute (AHRI) in Addis Abeba and frozen at −20°C until further processing.

The rapid test (RT), which is a colored latex-based lateral flow technology using a mix of selected *M. bovis* antigens including ESAT-6, CFP10, and MPB83 was used to detect specific antibodies in animal serum (Chembio Diagnostic Systems, Inc.) [22]. Results were read 20 minutes after adding the buffer solution to the sera. Animals were considered positive if a visible line appeared on the test area of the RT in addition to the control line. Animals were considered negative if only the control line was visible.

Mesenteric lymph nodes were processed and cultured separately, whereas submandibular and mediastinal lymph nodes were pooled together. Following homogenization and neutralization according to standard methods [23], sediments were inoculated on three different media slants: Löwenstein-Jensen media with and without pyruvate, and Middlebrook 7H11 medium supplemented as previously described [24]. The slants were incubated horizontally at 37°C for one week then vertically for an additional five weeks. Cultures were considered negative if no visible growth was seen after six weeks of incubation. Positive culture were stained according to the Ziehl-Neelsen method and examined under the microscope to select for acid fast bacilli (AFB) positive isolates.

Heat killed AFB positive samples were investigated by multiplex PCR [25], including primers specific for the *Mycobacterium* genus and the *M. tuberculosis* complex. Strains typed as belonging to the *M. tuberculosis* complex were further characterized by PCR using species specific RD4 and RD9 primers [26] and by spoligotyping as previously described [27]. Isolates not belonging to the *M. tuberculosis* complex but positive for the genus were sequenced over the 16S rRNA locus [28].

### Ethics statement

Ethical clearance for the human part of the study was obtained from the institutional (AHRI/ALERT; Ref. P004/04) and National Ethical Review Committees (NERC; Ref. RDHE/178-71/2006).

### Sputum collection and analysis

Sputum was collected from farmers in the settlements where livestock were BTB tested, after informed consent, which was verbal because of illiteracy of Hamer pastoralists, and if they were showing the following clinical signs (coughing, haemoptysis, recurrent fever, weight loss, night sweat, poor appetite and general weakness) for longer than one month.

Sputum was collected by a clinical officer responsible for the DOTS program at the local hospital in Turmi. Specimens were collected in the morning and kept in 50 ml sterile Falcon® tubes (Becton Dickinson Labwaren, Franklin Lakes, NJ. USA) at 4°C during transport, frozen upright at −20°C for a period of 2 weeks before being processed in the diagnostic laboratory at AHRI, Addis Abeba. Specimens were cultured and investigated by molecular methods as described above.

### Statistical analysis

Data were double entered in Microsoft Access, data sets were compared with EpiInfo (version 3.3.2) and analyzed with the software package STATA 10.1 (StataCorp, Texas, USA). Cattle and goat data were analyzed using univariate and multivariate logistic regression with random effect on settlements and with both the >4 and >2 mm cut-off for BTB positivity as outcomes. The results were expressed in odds ratio, 95% confidence interval for the odds ratio and p-values. A settlement-herd was considered positive if at least one positive reactor was present. Wildlife was considered positive if a *M. bovis* strain was isolated from tissue culture. People were considered to be infected with *M. bovis* if the agent was isolated in the sputum material.

## Results

### BTB in cattle

A total of 499 zebu cattle from 42 different owners were tested in 12 herds of the 12 settlements. The majority of the cattle were females (76%) and 73% of the animals were breeders (3 years and older). Animals with normal to muscular body condition accounted for 94.6% (N = 472) and only 27 animals were thin (5.4%).

BTB individual prevalence was 0.8% (CI: 0.3%–2%) when using the >4 mm cut-off and 3.4% (CI: 2.1%–5.4%) when using >2 mm cut-off. Herd prevalence was 33.3% (>4 mm cut-off) respectively 83% (>2 mm cut-off).

The univariate analysis showed no correlations between age, sex, body condition and the outcome of positive BTB tuberculin reaction (Table 2).

**Table 2:** Univariate analysis of cattle variables for BTB reactors (using 4 mm and 2 mm cut-off) and for MAC reactors, calculated with logistic regression and random effect on settlement.

			4 mm cut-off			2 mm cut-off			MAC		
Variable		Number (%) animals	Number of BTB reactors	p-value	OR (CI 95% OR)	Number of BTB reactors	p-value	OR (CI 95% OR)	Number of MAC reactors	p-value	OR (CI 95% OR)
Sex	Female	381 (76.4)	3			16			11		
	Bull	90 (18)	0	1.0	—	0	1.0	—	2	0.7	0.7 (0.2; 3.5)
	Ox	28 (5.6)	1	0.2	4.6 (0.4; 46.3)	1	0.8	0.8 (0.1; 6.6)	0	1.0	—
Age	Breeder	259 (52)	2			11			9		
	Calves	38 (7.6)	0	1.0	—	0	1.0	—	1	0.8	0.7 (0.09; 6.2)
	Juveniles	94 (18.8)	0	1.0	—	0	1.0	—	2	0.5	0.6 (0.1; 2.8)
	Old	108 (21.6)	2	0.3	2.4 (0.3; 17.4)	6	0.6	1.3 (0.5; 3.7)	1	0.2	0.2 (0.03; 2.0)
Body condition	Normal	27 (5.4)	2			14			11		
	Emaciated to thin	418 (83.8)	1	0.09	8 (0.7; 91.1)	1	0.9	1.1 (0.1; 8.8)	1	0.7	1.4 (0.2; 11.5)
	Musculous to fat	54 (10.8)	1	0.3	4 (0.3; 44)	2	0.9	1.1 (0.2; 5.0)	1	0.7	0.7 (0.08; 5.5)

### BTB in goats

A total of 186 adult goats underwent CIDT in four settlements. All animals had a good body condition and 70% were females. None of the tested goats were BTB reactors for neither the >4 mm nor the >2 mm cut off.

### BTB in wildlife

Fifty-one animals from thirteen different species were sampled. Nine out of 33 animals (27%) were sero-positive on the rapid test. High sero-prevalence was found in lesser kudu (*Tragelaphus imberbis*) (N = 3; 60%) and Grant's gazelles (*Nanger granti*) (N = 4; 33.3%). Twenty-four out of 48 (50%) cultures yielded AFB positive colonies. No *M. bovis* was isolated from the cultures and the great majority of the strains (79.2%) belonged to the *Mycobacterium terrae* complex (19/24 AFB +). Three Grant's gazelle and one lesser Kudu were positive in the rapid test and were NTMs (*M. terrae* complex, *M. flavescens, M. moriokaense and M. wollinsky*) (Table 1).

### BTB in humans

Sputum was collected in 4 settlements from 26 pastoralists (15 females and 11 males) with tuberculosis (TB) symptoms. None of the patients had been previously treated with antibiotics nor received BCG vaccination. Three households had positive BTB reactor-cattle with the >2 mm cut-off. Age of patients varied between 12 and 70 years. All 26 patients were coughing and 9 (34.6%) showed haemophysis (Table 3). The majority of patients (57.6%) mentioned symptoms having started less than 3 months ago. In 4 patients (15.4%) the symptoms were present since 3 to 6 months and in 7 patients (27%) they existed longer than 6 months. Four cultures out of 26 (15.4%) were AFB positive. One isolate was characterized as *M. tuberculosis* by deletion typing and spoligotyping. The other three were genus typed and yielded following NTMs: *M. conceptionense, M. senegalense, M. fortuitum and M. farcinogenes* (Table 3).

**Table 3:** Clinical signs and culture result of sputum collected from pastoralists in Hamer Woreda classified by age group.

Age group (year)	Number (%)	Cough	Haemophysis	Fever	Weight loss	Night sweat	Loss of appetite	Weakness	Culture positive	Culture result
0–14	1 (3.8)	1 (100)	1 (100)	1 (100)	1 (100)	1 (100)	1 (100)	1 (100)		
15–24	1 (3.8)	1 (100)	1 (100)	1 (100)	1 (100)	1 (100)	1 (100)	1 (100)		
25–34	5 (19.3)	5 (100)	2 (40)	4 (80)	4 (80)	4 (80)	4 (80)	4 (80)	1 (20)	NTM*
35–44	2 (7.7)	2 (100)	0 (0)	2 (100)	1 (50)	2 (100)	1 (50)	1 (50)		
45–54	4 (15.4)	4 (100)	1 (25)	4 (100)	4 (100)	4 (100)	4 (100)	4 (100)	1 (25)	*M. tuberculosis*
55–64	11 (42.3)	11 (100)	3 (27.3)	10 (91)	9 (81.8)	10 (91)	10 (91)	10 (91)	2 (18.2)	NTM*
65+	2 (7.7)	2 (100)	1 (50)	2 (100)	2 (100)	2 (100)	2 (100)	2 (100)		
Total	26 (100)	26 (100)	9 (34.6)	24 (92.3)	22 (84.6)	24 (92.3)	23 (88.5)	23 (88.5)	4 (15.4)	

NTM* included M. conceptionense, M. senegalense, M. fortuitum and *M. farcinogenes*.

### Mycobaterium Avium Complex (MAC)

Thirteen cattle out of 499 were skin test reactors for MAC, resulting in a prevalence of 2.5% (CI: 1.3%; 4.8%). Univariate analysis of cattle variables are shown in Table 2. None of the variables age, sex and body condition were associated with positive MAC reactors, neither in the univariate nor in the multivariate analysis.

One out of 186 goats was skin positive for MAC, resulting in a prevalence of 0.5% (CI: 0.07%; 3.7%). Two wildlife out of 48 (4.2%) were MAC positive on culture; both animals were Grant's gazelles (*Nanger granti*).

## Discussion

Multiple studies have been performed in the Ethiopian Highlands in recent years on BTB in cattle, which included urban/peri-urban areas with high concentration of dairy farms or rural areas characterized by traditional mixed crop-livestock small holder farms with small zebu herds [9], [11]–[13]. But little BTB research has been done in pastoral communities in the Ethiopian Lowlands although they account for 13% of the total population, live at the edge areas of the highlands covering 61–65% of the total country size [29] and own high number of cattle and goats. Livestock programmes (e.g. veterinary services, research, census) often omit these more mobile and/or remote communities.

In this study, the authors found an individual apparent overall BTB prevalence of 0.8% (>4 mm cut-off), which is much lower than the 5% prevalence described in Borana region by Demelash et al (2009) [14] and Gumi et al (2009) [15]. Individual prevalence ranged between 0% and 4% (>4 mm cut-off) and between 0% and 7.7% (>2 mm cut-off) in the different settlements, thus the disease seemed to concentrate on some particular spots (hot-spot sites), despite the high mobility and contact rate of livestock in the study area, which is in line with the findings of Gumi (2009) [15]. These figures show that the regional epidemiological assessment of BTB and disease prevalence has to take into account the high variability amongst villages.

Here, many animals were observed with enlarged prescapular lymph nodes prior to PPD injection, suggesting on-going underlying chronic diseases. Chronic debilitating co-infections, such as contagious bovine pleuropneumonia (CBPP), trypanosomiasis and endoparasites –all prevalent in the area according to local veterinarians- are likely to affect the CIDT result by down-modulating the animal's immunity and thus leading to increased false negative animals in this area and thus possible underestimation of the BTB prevalence. Exact prevalence of most of these chronic diseases in Hamer and their role in immuno-modulation by down-regulating Th1 response is unknown and more research is warranted on that subject.

Emphasis of national control programs of BTB is clearly on cattle herds, while small ruminants, particularly goats, are rarely included in these programs. *M. bovis* has been increasingly isolated from goats in various countries and showed often severe clinical symptomatic [30]–[32]. In our study site goats were twice as numerous as cattle (Woreda Agricultural office, Dimeka) and are an important source of meat protein in the Hamer population. No BTB reactors were found among goats in this study. However, this result needs careful interpretation. The method and interpretation of the intradermal testing is not officially standardized for goats and although the OIE suggests following the same procedures as with cattle, many authors used their own definitions for reactors with cut-offs for positivity varying between 2 and 4 mm, thus making comparisons of results difficult [32]–[34]. Technical and/or interpretation bias can therefore lead to false PPD negative animals. Furthermore, goats in Hamer were kept separately from the cattle herds (own enclosure at night, shepherded separately during the day) thus the contact rate between cattle and goats was minimal. In addition, their life turn-over is greater than cattle which could explain why BTB has no time to get established in a herd. Finally, PPD reactions in goats, as in cattle, might be masked by co-infections such as helminths and Contagious Caprine Pleuropneumonia (CCPP) that are the top caprine diseases in this region (Woreda Agricultural office, Dimeka).

Due to increasing environmental pressure (e.g. drought, decreasing grazing areas) livestock share the same pastures and water sources as wildlife (personal observation). However, in our study *M. bovis* was not isolated from wildlife tissue but the sample size was too small to be conclusive (n = 48). Consistent with studies in Tanzania, South Africa and Ethiopia [35]–[37], a high number of NTMs were isolated from wildlife tissue samples; with *M. terrae* complex being the most prevalent. In our study NTMs were isolated mostly from browsers suggesting that feeding behavior may play a role in the epidemiology. The high positive serology result has to be assessed with caution as the rapid test has not yet been validated for African wildlife species [22], [37]. It is possible that environmental mycobacteria may have cross-reacted with the test antigens leading to false positive sero-test results as suggested by four animals being positive for the rapid test as well as being culture positive for NTMs.

Two MAC strains were isolated from intestinal lymph nodes of Grant's gazelle. Although MAC prevalence was relatively high in wildlife (4.2%), it was lower in livestock (0.8% in goats and 2.5% in cattle). As shown in an abattoir survey in Jinka, that also slaughters surplus bulls originating from Hamer, 56% of all Mycobacteria isolates were NTMs including *M. fortuitum, M. gordonae* and *M. mucogenicum* [12]. NTMs were also isolated in three pastoralists showing clinical symptoms of TB, suggesting some degree of NTM pathogenecity in humans or overgrowing *M. tuberculosis*/*M. bovis* in culture. However, the *Mycobacterium* spp differed entirely from those NTMs isolated in wildlife. Our results suggest that even though livestock, wildlife and pastoralists were sharing intensively the same habitat, the prevalence of particular *Mycobacterium* spp seemed to be clustered to particular species.

Farcy is a serious chronic infectious disease in cattle caused by *M. farcinogenes* in East and Central Africa and *M. senegalense* in West Africa [38]. The authors cannot explain why these pathogens have been isolated in humans in Hamer although bovine farcy has never been previously described in Ethiopia. It is well possible that farcy is prevalent in cattle of pastoralists, but to date no publication exists on an isolate.

Despite the high number of individuals with TB symptoms, only one patient was bacteriologically confirmed. This discrepancy may be due to logistic constraints 1) only one sputum was taken from each patient instead of the WHO recommended 3 sputum samples [39] and 2) the long time between collection and processing of sputum samples. Both may have resulted in a decreased isolation yield of Mycobacteria isolation from culture.

In conclusion, this is to our knowledge, the first BTB study done in South Omo. BTB prevalence was low in cattle and non-existent in goats, wildlife and humans. In the contrary, NTM prevalence was relatively high in the region with a species-specific distribution. The role of these NTMs, as well as the role of co-infection in livestock with other infectious diseases prevalent in the region needs further research in terms of modulation of the animal immune system and thus its response towards the CIDT. Finally, this study highlights the lack of knowledge of the epidemiology of NTMs circulating between humans, livestock and wildlife in a same area.
